# Health shocks, care-seeking behaviour and coping strategies of extreme poor households in Bangladesh’s Chittagong Hill tracts

**DOI:** 10.1186/s12889-019-7335-7

**Published:** 2019-07-29

**Authors:** Ashraful Kabir, Rupa Datta, Sayeed Hasan Raza, Mathilde Rose Louise Maitrot

**Affiliations:** 1 Dushtha Shasthya Kendra, Dhaka, Bangladesh; 2 United Nations High Commissioner for Refugees, Dhaka, Bangladesh; 3 Caritas, Dhaka, Bangladesh; 4 Lecturer in International Development and Global Social Policy, Department of Social Policy and Social Work, The University of York, York, UK

**Keywords:** *Adivasi* (ethnic minority), Care-seeking behaviour, Bangladesh, Chittagong Hill tracts, Coping strategies extreme poor households, Health shocks, Qualitative study

## Abstract

**Background:**

How and whether health shocks, care-seeking behaviour and coping strategies are interlinked and influence households resilience to ill-health remains an under-researched subject in the context of Bangladesh. This study investigates whether and how health shocks, care-seeking processes and coping strategies interplay and impact the resilience of extremely poor *adivasi* (ethnic minority) households in the Chittagong Hill Tracts (CHT), Bangladesh.

**Methods:**

Our analysis draws from qualitative data collected through a range of methods (see Additional file 1). We conducted 25 in-depth interviews (IDIs) of two *adivasi* communities targeted by an extreme-poverty alleviation programme, 11 key informant interviews (KIIs) with project personnel (community workers, field officers, project managers), community leaders, and healthcare providers, and 9 focus group discussions (FGDs) with community members. Data triangulation was performed to further validate the data, and a thematic analysis approach was used to analyze the data.

**Results:**

Health shocks were a defining characteristic of households’ experiences of extreme poverty in the studied region. Care-seeking behaviours are influenced by an array of cultural and economic factors. Households adopt a range of coping strategies during the treatment or care-seeking process, which are often insufficient to allow households to maintain a stable economic status. This is largely due to the fact that healthcare costs are borne by the household, primarily through out-of-pocket payments. Households meet healthcare cost by selling their means of livelihoods, borrowing cash, and marketing livestock. This process erodes their wellbeing and hinders they attempt at achieving resilience, despite their involvement in an extreme poverty-alleviation programme.

**Conclusions:**

Livelihood supports or asset-transfers alone are insufficient to improve household resilience in this context. Therefore, we argue that extreme poor households’ healthcare needs should be central to the design of poverty-alleviating intervention for them to contribute to foster resilience.

**Electronic supplementary material:**

The online version of this article (10.1186/s12889-019-7335-7) contains supplementary material, which is available to authorized users.

## Background

Health shocks and poverty are deeply related [1, 2]. Health shocks defined as ‘unpredictable illnesses that diminish health status’ [3] are often recognized as a determining factor for poverty. Ill health and heath shocks in particular, often induce severe vulnerability. Research and data across international contexts provide evidence that health shocks lead to income and expenditure uncertainty that triggers impoverishment at the individual and/or households level [4]. For example, studies conducted in Cambodia, Ethiopia, Haiti, Sierra Leone, Senegal, and Vietnam indicate that healthcare costs negatively affect households’ welfare and can lead people into poverty as a higher proportion of treatment costs are borne by the households and paid through out-of-pocket expenditures [5–8]. Likewise, studies conducted in Bangladesh show that catastrophic health expenditure (CHE) leads to impoverishment and pushes households into poverty. For example, Alam et al. shows 3.5% of the total population (corresponding to approximately 5 million people) in Bangladesh fall into poverty annually due to out-of-pocket (OOP) payment mechanism wherein 16.5% of poorest and 9.2% of the richest households faces CHE [9]. Another study shows that households spend 11% of their total budget on healthcare wherein 9% households faced financial catastrophe. Further, this study shows the poorest have four times higher risk of catastrophe than the richest group [10]. Hamid et al. shows that annually 3.4% households are pushed into poverty due to OOP outlays wherein chronic non-communicable diseases are the principle contributor [11].

A common assumption is that when people experience health shocks, regardless of their magnitude, they all seek treatment; however, studies show that seeking treatment is a complex process, which can vary across demographics and geography. Treatment-seeking behaviour is shaped by a range of factors including age, sex, religion, ethnicity, household resources, costs of care, severity of illness, availability of health service, and access [12]. Health is correlated with socioeconomic conditions [13] that determine choice for and attitude towards particular treatments [14]. Grundy et al. [8] identified three health determinants—institutional and system, socio-cultural, and individual/household. Here, we also find that the extreme poor tend to be overexposed to health shocks and often endure painfully long, expensive and inefficient health-seeking processes, relying heavily on their little savings and energy [12, 14, 15].

Yet, over the past two decades Bangladesh has made unparalleled progress in some selected socioeconomic and health indicators. As far as economic performance is concerned, the country has maintained an annual GDP growth of over 6% over the past 15 years. Poverty and extreme poverty rates have significantly dropped– from 53% in 1995–96 to 24.6% in 2016 for poverty, and from 40 to 12.9% for extreme poverty [16, 17]. With respect to health, Bangladesh made remarkable progress in reducing maternal mortality, increasing infant and child survival, life expectancy, widening the coverage for contraceptive, immunization, and rehydration therapy [18].

Healthcare services in Bangladesh are delivered through various channels including public health departments of the government, private institutions, and NGOs [19]. The Ministry of Health and Family Welfare (MoHFW) is the apex body responsible for policy and program formulation, execution, management, coordination, and regulation of health, nutrition, and population related activities [20]. The country has developed a comprehensive health service delivery infrastructure with a vast network of primary health care facilities from grassroots to higher levels. Distribution of public health services follows uniform patterns of administrative tires—national to community levels. The international public health community applauded these health gains and tends to explain that the country has established and maintained an effective and well-functioning health system with limited resources [21]. However, these advances are experienced unequally across the population, often leaving behind individuals and communities that are economically marginalised and geographical dispersed. Improved health services, especially those provided by the state, are not yet effectively distributed to all individuals and groups, and frequently fail to reach ethnic minorities, people living in remote locations, extremely poor individuals, and other marginalised groups [19].

Extremely poor *adivasi* (ethnic minorities) communities in CHT^1^ for example have comparatively poor health outcomes and face challenges in accessing healthcare services because of dominant presence of informal providers (traditional healers), gender preference in accessing healthcare and decision making process (males get preference in seeking care), overriding cost, unitary service delivery mechanism (the current healthcare delivery system is predominantly based on the priorities and needs of plain land people), and inadequate knowledge and awareness [22] Over 2 million *adivasi* are Bangladeshi citizens. They are mostly involved in shifting agriculture which is also known as (*jhum*) in Chittagong Hill Tract (CHT) [23]. Their forest-based livelihood, language, cultural practice, religious faith and rituals are distinct from plain land Bengali’s [23, 24]. Since they largely reside in remote and peripheral area (mostly Chittagong Hill Tracts, CHT henceforth) where socioeconomic development tends to be lower and improving at a slower rate than in plain land, and the medical systems and health services remain problematic for the region’s development [24]. Consequently, the incidence of health shocks has been identified as central to *adivasi* groups’ livelihood resilience [23]. In the context of such poverty, livelihood interventions are promoted as a sustainable tool for poverty reduction and for improving the living conditions of disadvantaged groups. Livelihood interventions are generally deemed to positively impact poor households’ livelihood through increasing the assets and life skills of the individual and/or household. As such aforecited studies however also indicate, wellbeing and resilience of the households are affected by a high exposure to health shocks, long and costly care-seeking process, and coping strategies. This raises the question of how such livelihoods interventions impact on health seeking behaviour. In a previous study a number of the authors here explored how and whether health shocks impacted anti-poverty interventions [25]. Given the limited literature, in this study we focus on ethnic minority communities who are living in sparsely dispersed locations. Existing scholarship lacks information about the process through which health shocks, care-seeking processes, coping strategies interplayed and impacted on extreme poor *adivasi* households’ resilience to extreme poverty in the CHT area. This study therefore contributes to addressing an information gap in research on poverty and offers a strong body of evidence for policy planners, programme managers, and implementers to design effective poverty-alleviation programmes targeting ethnic minorities. This study, we hope, will contribute towards better extreme poverty-alleviation programming in Bangladesh and beyond.

## Methods

### Study time and settings

This study was conducted between May and September 2015 in three *upazilas,* namely *Lama*, *Naikhongchhari*, and *Ruma* of *Bandarban*—a Chittagong Hill Tract (CHT) district in Bangladesh. *Bandarban* is considered to be the most remote district of Bangladesh because of its geography—hills and rivers combined with poor road and communication infrastructures. These three *upazilas* share common boundaries and although they are characterized by various degrees of remoteness they together embody distinct socio-economic and geographic features compared to the rest of the country.

Lama is the biggest *upazila* of *Bandarban* and houses nearly half of the district’s population. The size of the *upazila* is 671.84 km^2^ of which 332.827 km^2^ is reserved forests [26]. The total population is 113,413 according to the census of 2011. There are six *adivasi* groups in the upazila—*Chakma, Marma, Murang, Tropura,* and *Tabjhong*. The major livelihood is agriculture (63%), and labouring (15%), services (8%), and animal husbandry and fishing (6%). The average literacy rate is 31% [27].

*Naikhongchhari* is 469 km^2^ [27], and according to the 2011 census, has 49,465 inhabitants. The major *adivasis* are *Chakma, Marma, Murang,* and *Tabjhong*. Their main livelihood is agriculture (51%), labouring (13%), transport and communication (9%), services (2%) animal husbandry and fishing (6%). The average literacy rate is also 31% [27].

Ruma is has a total population of 2, 02,683, according to the 2011census. Eleven *adivasi* groups reside in this *upazila* [26]. The major groups are *Chakman, Marma, Murang, Tropura*. The primary livelihood is agriculture (85%), labour (13%), transport and communication (6%), services (2%) animal husbandry and fishing (6%). The average literacy rate is slightly lower than in the other two *upazilas* at 26% [27].

### Project intervention

In order to support the Government of Bangladesh’s (GoB) efforts to eradicate extreme poverty and hunger by 2015 (Millennium Development Goal 1) a programme called ‘Economic Empowerment of the Poorest/Stimulating Household Improvements Resulting in Economic Empowerment (EEP/Shiree)’ was developed through a partnership between the GoB, the ‘UK Department for International Development (DFID) and the ‘Swiss Agency for Development and Cooperation (SDC)’. The programme ran between 2008 and 2016. Its aim was to lift 1 million extremely poor people out of extreme poverty and improve their resilience. To achieve this objective, EEP/Shiree introduced two separate categories of funds—a ‘scale fund’, and an ‘innovation fund.’ The scale funds were provided to NGOs judged to have the capacities to facilitate large-scale interventions with tested and well-established models of intervention; while, innovation funding were awarded to innovative approaches to reducing extreme poverty in Bangladesh. The project under study in this paper, “Ensuring Sustainable Livelihood of Extreme Poor of Chittagong Hill Tracts”, came under the remit of the innovation fund and was implemented by Caritas Bangladesh (ESLEP-CHT) in five *upazilas*.

### Participants and sample strategy

We draw our analysis from interviewing *adivasi* participants from the ‘Ensuring Sustainable Livelihood of Extreme Poor of CHT (ESLEP-CHT)’ project and project staff responsible for the intervention’s implementation (Table 1). We conducted in-depth interviews (IDIs) with individual beneficiaries from the ‘*Marma’* and ‘*Murang’* ethnic groups from three *upazilas*. We also carried out Key Informant Interview (KII) in Lama, and *Ruma* —3 were conducted with members of the implementing NGOs (Caritas Bangladesh), 4 with community members including a teacher, religious leader, and local government representative, and 4 with formal (govt.) and informal healthcare (traditional) providers. Additionally, we carried out 9 Focus Group Discussions (FGD), 4 with community members and NGO field staffs, 5 with community members. A purposive sampling strategy was used in recruiting participants—we considered three inclusion criteria—age > 18 years old, willingness, and time availability. We also ensured maximum variation and gradual selection (A sampling strategy in qualitative research where a diverse range of participants are selected following an iterative approach to uncover central themes and dimensions) of participants. The participants were questioned on their past experiences of health shocks, their care-seeking and coping strategies, and the consequences of these on their lives and livelihoods. The sample size was determined according to the principle of data saturation—at a point where the researchers notice no new information and/or theme and/or dimension emerged in the interviews [28], it was considered that the amount of data was sufficient.

**Table 1:** Data collection methods and respondent characteristics

In-depth interviews (IDIs) [*n* = 25]	Participant characteristics	Ethnic communities		Upazila
		*Marma* (*n* = 14)	*Murang* (*n* = 11)	
	Age in years (mean ± SD)	27 ± 8	29 ± 7	*Lama, Naikhongchhari*, and *Ruma*
	Monthly household income in BDT (mean ± SD)	4300 ± 550	4600 ± 580	
	Sex (*n*)			
	Male (*n = 16*)	9	7	
	Female (*n = 9*)	5	4	
	Education (*n*)			
	1–5	3	4	
	6–10	2	2	
	10+	1	0	
	No formal schooling	8	5	
	Marital status (*n*)			
	Married	9	6	
	Unmarried	5	5	
	Family Type (*n*)			
	Extended	6	3	
	Nuclear	8	8	
	Occupation (*n*)			
	Jhum cultivation	7	3	
	Handcrafter	1	2	
	Small business	2	1	
	Day labor	2	3	
	Hunting and gathering	1	1	
	Others	1	1	
	Religious belief (*n*)			
	Buddhism	11	8	
	Others	3	3	
Key Informant Interview (KII) [*n* = 11]				
	3 representatives of implementing NGOs	4 community leaders including, teacher, religious leader, and local govt. representative	4 healthcare providers including govt. and traditional healers	*Lama*, and *Ruma*
Focus Group Discussion (FGD) [*n* = 9]				
	4 including Community members, and NGO field staffs	3 Community members	2 Community members	*Lama, Naikhongchhari*, and *Ruma*

### Data collection procedure

A team of 3 interpreters and 3 researchers graduated in anthropology and public health conducted interviews and discussions in the field. The team members received significant training on qualitative research and had extensive field experiences researching poverty and NGO interventions. Interviews and FDGs were conducted in Bangla with the NGO staff and *adivasi* languages *Marma*, and *Mru* with participants. A semi-structured interview guideline was used to cover a range of topics relating to health shocks, and care-seeking process, and their consequences on livelihoods in the context of extreme poverty. On average each IDI and FDG lasted between 50 and 65 min and 90 to 120 min, respectively. Before commencing conversation, the researchers established good rapport with the interviewees. Detailed field notes were taken during the conversations and all interviews were recorded, transcribed verbatim, and subsequently translated into English. In some cases, follow-up visits were arranged to fill gaps and to probe some early findings.

### Data analysis

Thematic analysis was used to explore the interviews’ and group discussions’ transcriptions [29]. Firstly, we generated codes collectively through repeatedly reading the data, and then coded all transcripts. Secondly, having completed the initial coding of the interviews, we independently looked for clusters of several codes—termed “themes” or “concepts.” Thirdly, to increase the validity of the coding system, as the research team members discussed emerging themes and early findings they triangulated the information collected before reaching a consensus on core concepts. Software for textual analysis such as *ATLAS-ti*, and/or *Nvivo* were not used to organize or code the data.

### Ethics

This study obtained ethical approval from the University of Bath, UK, a member of the EEP/Shiree management consortium responsible for overseeing the qualitative research undertaken through the programme. Locally, we obtained ethical approval from ‘Stimulating Household Improvements Resulting in Economic Empowerment’, a development programme under the Rural Development and Cooperatives Division (RDCD) of the Ministry of Local Government, GoB. We developed an informed consent paper to explain the objectives, importance, anonymity, confidentialities, possible risks and benefits, participant’s right and potential source of further information. A written consent was read out and encouraged the participants to query the interview process. We sought written approval before conducting each interview, and documented the interviews/discussion via audio recording. We removed the names of interviewees during data analysis and used codes to anonymise the data.

## Results

This section briefly presents the 25 participants’ demographic profiles (Table 1) and provides details about the KIIs and FDGs. The mean age for ‘*Marma*’ was 27 (SD ± 8), and 29 (SD ± 7) year for ‘*Murang*’. Among them, 14 were male and 11 females. More than half of participants had no formal schooling, while 1 had X grade schooling. The large majority of participants were married and belonged to a nuclear family structure (16). The predominant occupation was *jhum* cultivation (10), followed by day labouring (5), handicraft (3), and small business (3).

**Fig. 1:**
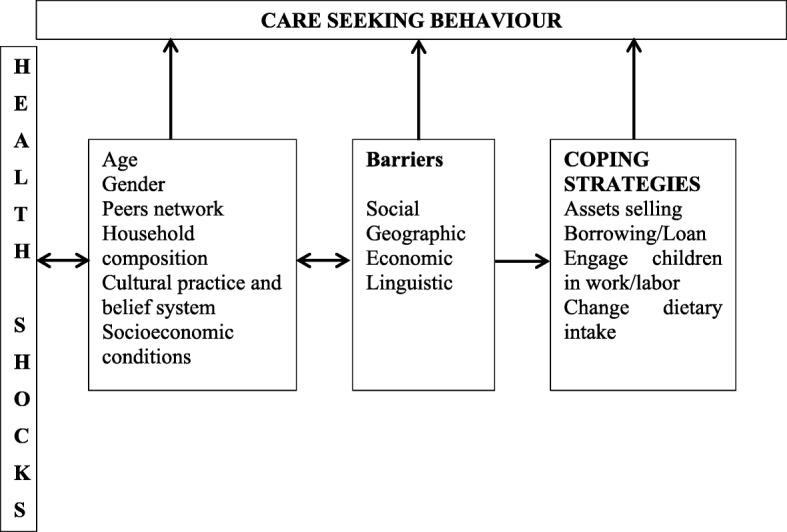
Dominant themes emerged

Based on the initial ‘code’ we categorised them into three themes (Fig. 1) which are interrelated and influence each other.

### Health shocks

All the participants reported having experienced multiple health shocks during the project intervention. In particular, they reported many short-term shocks that varied with seasonality and chronic illnesses. For example, during the summer season, communicable disease and infection i.e. diarrheal diseases, malaria, and typhoid were prevalent in all three *upazilas*. In the winter period, participants reported respiratory and skin diseases such as pneumonia, asthma, scabies, eczema, itching and skin allergy, and fever to be common. Non-communicable Chronic Diseases (NCDs) including diabetes, cardiac complications, and maternal illnesses, were commonly reported from all sites. A female NGO employee stated:

*‘We noticed that many project beneficiaries suffer from communicable disease in the monsoon. […] but in the winter mostly they face respiratory illness like asthma, and common cold. Other non-communicable diseases appear to be prevalent throughout the year.’*

### Health seeking behaviour

Care-seeking behaviour was influenced by a range of factors including age, gender, peer network and influence, cultural practice and belief system, as well as the socio-economic status of the individuals and/or households. Participants commonly reported that decisions regarding individual care were taken by the household head, which often is a working-age male household member. Females, children, and other non-income-earning household members (disabled or very elderly) are more likely to be taken to informal healthcare providers like homeopaths or traditional healers than working male members. Their care-seeking process is also likely to be neglected and/or delayed. One male participant reported this directly during a focus group discussion:

*‘Men are the key earners and mostly the decision maker of the family; because of this they have the most influence over the health seeking process. […] women and children are given less attention in seeking care within the family.’*

This participant also explained that the care-seeking process tended to start quickly after the first signs of illness of income-earners who by and large are male household members. Among others, senior members of the households for example parents and in-laws were found to be influential in the care-seeking process. As one participant explained.

*‘Parents are respected. Besides, they may hold the ownership of property; […] they normally get good care from family members.’ (A male participant from IDI in Naikhongchhari).*

Peers and neighbouring networks, religious beliefs and rituals appeared to influence the care-seeking process significantly. Some participants viewed neighbours’ roles as useful when seeking care. A participant explained this process during an in-depth interview:

*‘Sometimes, neighbours come up with good advice about seeking healthcare. It might be helpful to get useful information about what type of care a person needs and possibly where from.’ (A male participant from IDI in Lama).*

However, nearly one-fourth of participants reported that neighbour rarely show interest while a person is ill. Rather, it is considered a private matter whether to seek care or not and how to do so.

Religious beliefs and rituals shaped the care seeking process as a few participants claimed those people who follow their religion strictly are likely to seek care from religious healers. Households often perform group prayers and regular offerings to their God (sacrificing livestock). These practices incur significant additional expenses as one participant’s experience shows:

*‘I sacrificed one goat, one pigs, and nine chickens which cost large amount of money.’ (A female participant from IDI in Naikhongchhari).*

Elderly members of the household are likely to have a preference for care from traditional healers. As one of the participant reported:

*‘The elderly people might have been used to taking traditional care throughout their generation. And they still opt for their service. […] the younger generation is often not so convinced by them.’ (A female participant from FGD in Naikhongchhari).*

Socio-economic conditions were commonly mentioned to be a strong determinant of the course of seeking care. For the extreme poor, informal care providers such as *Baiddaya* (herbalist) and religious charmers were common first points of contacts. Their services incur relatively low costs and are believed to be efficient for major common illness. A participant reflected:

*‘Baiddayas (traditional herbalists) are knowledgeable and good at giving you useful care. Many of us get cured through their treatment’ (A female participant from IDI in Ruma).*

Moreover, many participants reported that traditional herbal medicine is a central element of their local health system. People who suffer from skin problem like itching, allergy, eczema, fungal infections and scabies rely on herbalists. They however are not considered as efficient when it comes to treating chronic diseases. An experienced male NGO staff member said:

*‘Herbal medicine and herbalist is a strong element of local health system. Possibly, it can bring some good results in case of common illness. But, it might not be effective for chronic diseases which force families to be trapped in poverty.’ (from KII in Lama).*

In addition, self-medication was identified as a common first step in the health-seeking process of the extreme poor across all sites. Simultaneously, unqualified allopath practitioners are popular because they are easily accessible with low cost. But the problem is that many times it leads to complication and mistreatment which result in higher health risk and larger cost.

### Barriers

Geography was reported to be a significant barrier to seeking care from formal (public) healthcare services. Mobility is challenging and people rely on local jeeps, rickshaw, buses, or boats to travel. When someone faces a health shock, regardless of whether the medical facilities exist, travelling to the place of treatment is a significant obstacle, often discouraging people from going to a hospital or visiting qualified doctors. One participant stated:

*‘I live six kilometres from Ruma town. When I fractured my leg I could not find a way to travel there as it involved crossing the hills and rapid rivers to reach the nearest hospital. There was no qualified doctor nearby, nor was there a paraprofessional or local medicine seller could access to get a medical check-up. I therefore went to see a traditional healer and religious charmer both of who were near her village.’ (A male participant from IDI in Ruma).*

Structural obstacles such as a shortage of healthcare providers, irregular supply of medicine and equipment, limited investigation and diagnostic facilities, and general poor quality of services in *Upazila Health Complex* (the first-line hospital in Bangladesh) was reported as a barrier from all sites and participants. A participant explained.

*‘Currently there are only four doctors in Ruma upazila, whereas there are thirteen available posts. Lama and Naikhangchari Upazilas have three and two doctors [respectively] whereas eleven and twelve posts are available.’ (A male participant from KII in Ruma).*

Similarly, another percipient explained:

*‘Critically ill people were carried on people’s shoulders to the Upazila Health Complex and returned to their villages without receiving proper treatment because there was no medical officer there.’ (A male participant from KII in Lama).*

Furthermore, negative experiences with formal health facilities confirmed communities’ prejudice against modern and/or public medical treatment. A participant reported:

*‘A new-born baby got sick and died after getting an immunization shot. That resulted in people losing faith on vaccination among Marma community as they think the immunization caused the baby’s death.’ (A male participant from FGD in Ruma).*

Apart from these, some participants reported that they faced linguistic problem to explain their complication as some the healthcare providers are unable to understand the patients’ language. This problem was reportedly severe when the healthcare provider is not from an ethnic minority.

### Coping strategies

#### Assets selling/breaking savings

Our data revealed that households adopted two strategies to cope with health shocks—firstly, to increase income or money flow in the household and secondly, to reduce expenses. These processes are often long and involve immediate and/or longer-term costs. Sometimes the household use their little savings; at other times, they sell their property and/or take loans. Household mobilize resources, often having to sacrifice their wellbeing, assets, time, and labour. However, the extreme poor households in the CHT have a very low propensity to save. It was revealed that there is no scope for them to save formally through institutions in (there are few banks and few microfinance institutions in *upazila* town). Extremely poor households tend to save little through informal savings systems. Occasionally, households save for a particular purpose (house repairs or other livelihood assets), but rarely for unpredictable emergencies. In some cases, the small amount of money saved is used for seeking a cure for mild illness of a household member but often the amount cannot cover the costs of serious illnesses. A participant stated:

*‘I had saved BDT 3000 [(US$ 37), considering BDT81=US$1] to repair and rebuild my house, but I could not do so because I had to use BDT 2000 (US$25) for my mother’s, my daughter’s, and my own treatment.’ (A male participant from IDI in Lama).*

Furthermore, unsuccessful treatments combined with high costs (direct and indirect, financial and human costs) and time consumption worsens the situation of extreme poor households who generally keep seeking various types of treatment until cash and savings run out. Sometimes they wait for the harvest season to be able to try again. When households have little to no cash savings to afford treatment, or when they are searching for effective treatment options, they tend to mobilize financial resources by selling or mortgaging assets. Although this enables them to gather cash to cover health related costs, selling and using physical assets (productive or non-productive) often has long-term impacts on a household’s resilience. Commonly, households sell livestock (if available) or fruits, crops, spices, and vegetables that were initially kept for their consumption. One participant stated:

*‘Caritas gave me two pigs. I have reared them. But, I had to sell one to manage money. If I hadn’t sold it for treatment costs, I could have reared it for longer and sold it at a higher price. I could have used the money to buy more pigs or some other asset.’ (A male participant from IDI in Naikhongchhari).*

Sometimes households sell input supports provided by their NGO since selling an asset makes up for an immediate liquidity shortage. On the other hand, it often impoverishes them further in the long run as they are compromising future financial returns and/or well-being. A sufferer stated:

*‘While trying to cure a life-threatening disease (cancer tumour), we were forced to sell our assets and take out a loan. However, we did not receive good treatment from the specialized doctor. We ended up assetless and unable to work, forcing my family into destitution.’ (A male participant from IDI in Ruma).*

### Borrowing/loan

In cases where extreme poor households do not have savings or assets (or not enough of these), or where they have exhausted these resources to afford effective treatment, they often resort to borrowing money. There are a few informal sources of loans available such as relatives and friends, landowners, moneylenders, community-based societies and rarely Microfinance Institutes (MFIs). Borrowing from neighbours and relatives is generally preferred because although the available amount is small, the interest is generally low compared to other options. Generally, patients during this time have little scope and borrow at high interest rates from moneylenders and employers who generally are the richest households in the community. These loans are expensive for the extreme poor (often 50% annual interest rate) but they are available throughout the year and repayable after the *jhum* cultivation season. If they fail to repay, then they often have the opportunity to repay during or after the harvesting season (applying another 50% interest on the loaned amount). An IDI participant stated:

*‘I took BDT 20,000 (US$ 247) from my sister (with no interest) and BDT 15,000 (US$ 185) from Karbari (a community leader) at a 5% monthly interest rate. As a result, I have accumulated a lot of debt. It can be more than BDT 50,000 (US$ 617) which I struggled to repay due to my inability to provide physical labour.’ (A male participant from IDI in Naikhongchhari).*

However, Caritas’ social safety-net support provides some help to extreme poor beneficiaries facing health shocks. If patients can contact and inform respective Caritas staff members of the ‘EEP/Shiree’ project; they can receive BDT 1000 (US$ 12) for primary treatment costs, during the project lifetime.

### Engage children in work/labour

Most of the time coping strategies of extremely poor ill health sufferers directly affect children’s wellbeing and education. While households struggle to accommodate the expense of treatment, they start cutting back on daily expenditures. One of the areas of compromise is children schooling. To increase their earnings and reduce expenses, participants reported that households often withdraw children from school and (if the child is old enough) engage them in labouring activities (paid or unpaid). They often work on their own *jhum* land. A participant stated:

*‘My daughters were engaged in jhum cultivation because of my illness. I had taken out a huge loan and without a good yield I would not be able to pay it back. As a result, my daughter had to work.’ (A male participant from IDI in Lama).*

Similarly, another participant reflected.

*‘My 13 years old son studied in grade III, but is now working on our jhum land.’ (A male participant from IDI in Ruma).*

Children thereby contributed to the treatment of the extreme poor income-earner. This mainly happens in cases of long-term illness or disability, and is less frequent in cases of short-term sickness.

### Change dietary intake

Participants reported that their households significantly compromised on the quantity and quality of the food they consumed in an attempt to reduce their daily expenses, mobilize more cash savings and meet healthcare costs and repay loans. They took less protein (fish and meat are expensive) intake and preferred to collect vegetables from nearby fields and forests. A participant said:

*‘I had BDT 500 (US$ 6) cash in hand to buy food for my family. When I was attacked by a viral fever, I spent BDT 120 (US$ 1.5) to buy medicine. I had to readjust the expenses by cutting food consumption.’ (A male participant from IDI in Lama).*

Another participant echoed:

*‘I am worried about the future. If we eat more, our rice stock will run out early and then we might need to borrow. So, it is better to reduce our daily food intake and save for future days.’ (A female participant from IDI in Naikhongchhari).*

### Resilience

Participants reported having little ability to mobilise sufficient funds for treatment from their savings. Most of the time, they use their cash savings for daily expenditures. As a result, they compromise on daily food consumption and children-related expenses. Such healthcare expenses diminished the potential for improving lives and livelihoods (e.g., home repairs, buying livestock, cultivation, education and local/religious festivals). Using that money for medical purposes prevents them from doing what they had planned. A participant remembers:

*‘If I had not spent money on treatment, I could have repaired my house. But illness left no other choice except suffering during monsoon. Now rainwater will pour through the holes in the roof. To cope with ill health, I have compromised on my housing’ (A male participant from IDI in Naikhongchhari).*

Another participant echoed:

*‘I am worried about the future. If we eat more, our rice stock will run out early and then we might need to borrow. So, it is better to reduce our daily food intake and save for future days.’ (A male participant from IDI in Ruma).*

Compromising on food consumption has long-term impacts on the nutritional status of all household members, including the sufferer’s. Taking fewer meals and lower quality food (less diverse and less nutritious) reduce household members’ short-term labouring and earning capacity, which in the long-term weakens their physical and mental health, consequently putting household members at risk of being afflicted by illness more often.

Typically, when the income earner becomes unable to work, young household members and wives would quickly adjust their livelihood and compromise their wellbeing. Their reproductive and care work at home, and work outside the homestead tend to increase in order to help the household cope. While women spend increasingly more time working in the fields, they still remain in charge of most household chores (cooking, taking care of children, and so on). Increased labour adds extra workload on household members and hampers ‘normal life’. As a participant expressed it:

*‘I cannot think of marrying my daughter because she has replaced me as the main income-earner.’ (A male participant from IDI in Lama).*

Overall participants reported that health shocks decreased households’, individuals’ and/or communities’ ability to anticipate, cope with, and recover from adversity, adversely affecting their long-term plans and prospects.

## Discussion

This study aimed to understand health shocks experiences, care-seeking behaviours, coping strategies and their implications for the resilience of extreme poor *adivasi* households living in the CHT and benefiting from a poverty-alleviation intervention. The factors identified, emerging from multiple qualitative data sources, are largely comparable, are interconnected and influence each other (Fig. 1). Health shocks were frequently reported throughout the interviews causing larger healthcare expense, which was made via out-of-pocket payments.

An earlier study [30] conducted in CHT shows that major illness included fever, diarrheal disease, under-nutrition, influenza; however, in our study chronic non-communicable (NCDs) diseases were those reported to incur larger cost. Although in this study we did not estimate the prevalence of NCDs because of the qualitative nature of the study design, the participant households were severely affected by the NCDs because they need to take prolonged facility based care. Our findings show that an epidemiological transition is underway where NCDs are coming forward in Bangladesh [31, 32].

In line with previous studies, our study reveals that informal healthcare providers were predominant in the care-seeking process [30, 33, 34]. For example, homoeopaths, and unqualified allopath were reported as a primary point of contact in this process. Furthermore, gender and age appeared as determining factors. Women and adolescent girls tended to seek care less frequently and later than men, a finding which is supported by earlier studies [15, 30]. Informal care providers are usually chosen for the treatment of children and women; however, considering the perceived severity of illness such as chronic condition (serious injury, cardio-vascular problem, congenital complication, tumour), and severe infectious disease (malaria, typhoid) they sought care from qualified health care providers (qualified allopath) [35]. Additionally a higher proportion of participants chose self-medication because it incurred little and/no cost which is concordant with other observation [36]. Cultural and religious believes, traditional practices and rituals strong drivers in the care-seeking process, such that traditional healers, religious charmers, and herbalists were very popular among the community, possibly because this process was of relatively low cost [33, 34]. Further, geographical marginalisation reinforced households’ preference for traditional healers, religious charmers, and herbalists as quality care especially from qualified healthcare providers was deemed more removed and thereby less accessible. Unlike other studies, our finding shows socio-economic [37] status of the household appeared as determining factor for choosing providers. In line with studies across global context, our study reveals that language skills reported to be a major inhibiting factor for seeking care from the formal health service as it jeopardized effective communication between the service users and health care personnel, making this services less convenient and accessible [38–41]. In our study the context was even more complex as fourteen [24] major ethnic minorities who maintain distinct language live in the CHT.

Our study show that high occurrence of health shocks severely affect the economic status of EEP/Shiree beneficiaries and their prospects [25]. Out-of-pocket payment system coupled with high opportunity costs resulted in extreme poor households being unable to pursue their livelihood strategy and in many cases caused them to fall deeper into extreme poverty. Studies across global contexts are in line with this finding wherein the poor household covered direct and indirect healthcare cost and subsequently forced to deeper poverty [42, 43]. A survey study confirms that ill-health and poverty are closely associated and they maintain a causal direction wherein poverty produces ill-health, which then sustains poverty [1]. Another study conducted by van Doorslaer [44] estimated that the absolute rate of poverty is 14% higher that the figure estimated using a conventional method which does not include out-of-pocket payment for using health services. This study further argued that there is an estimated 3.8% increase in poverty caused by healthcare related cost in Bangladesh [44]. However, some studies argue that households’ economic status is determined by a set of factors. For example, Krishna and colleague shows in a trajectory study in India wherein 85% of all cases declined into poverty caused a combined factors that included healthcare expense, private debt with higher interest rate, and social and customary expense [45].

In response to health shocks the households adopted a range of coping strategies (savings, asset selling, loans, and food consumption). The studies conducted globally support our finding. For example, Quintussi and colleague showed 34% of poor household in India are affected by health shocks and cope with such adverse events through selling assets, dissaving, and borrowing [46]. Similar observation were made in other studies such as taking credit from money lender, relatives, and friends in Cambodia, Indonesia and India [47–49], cutting food consumption in Laos [50] and selling livestock in Burkina Faso [51].

The immediate impact of health shocks was very significant as it affected income earners in most cases. The key income earners lost their livelihoods which generally affected nutrition, savings, children’s education, and child labour, food consumption—and in the end, their households’ resilience to poverty. Although resilience to poverty varies significantly according to the severity of diseases, generally health shock diminished households’ potential for overcoming poverty and exacerbate their vulnerability. The coping strategies analysed in this paper also suggest that this processes has inter-generational implications for the prospects of *Adivasi* households’ children*.*

### Limitation of the study

The findings of this study were elicited from a small sample size because of qualitative study design.. To limit the possibility that this biased the findings we triangulated our findings using a number of qualitative research tools (KIIs, FGDs), and through reference to wider available literature. We thus presented a detailed and in-depth account of factors that interconnect and are influence each other in the context of health behaviour in the CHT. We therefore believe this study presents a fair understanding about health shocks, care-seeking behaviours and coping strategies among ethnic communities in the CHT. Given that the focus of this study is on ethnic minorities in a geographically unique region of Bangladesh, findings will in certain regards likely not be generalizable to the majority Bengali Muslim population living in the plain lands.

## Conclusions

The findings of this study argue that health shocks are a common phenomenon among extremely poor *adivasi* households in the CHT. A number of complex factors made the treatment-seeking process difficult and as a result the extreme poor lack access to adequate medical care and the treatment seeking process is lengthened. The households adopted different coping strategies during the treatment process which were often not sufficient to allow households to maintain a stable economic status, and often this status in fact declined because of these strategies. This is primarily due to the out-of-payment mechanisms by which the majority of healthcare costs are borne by households. Having no alternatives, extreme poor households meet medical costs by selling their means of livelihoods, borrowing cash, and marketing livestock leading them to erode their labouring capacity and economic resilience despite the poverty-alleviation intervention. A key conclusion drawn from this study is that livelihood support alone is insufficient to protect the wellbeing and socio-economic status of extreme poor households. Therefore, we argue that households’ high exposure to health shocks, limited access to formal sources of medical care, and limited coping capacity ought to be taken into account in the design and implementation of poverty-alleviation programmes.

## Additional file

Additional file 1: of Health shocks, care-seeking behaviour and coping strategies of extreme poor households in Bangladesh’s Chittagong Hill TractsS1_Interview_Guidlines. (DOCX 23 kb)

## Data Availability

As we informed the participants during the consent process that data would only be shared within the research team, then the data cannot be made available publicly. Interested parties may contact Mr. Md. Mirazul Islam, Administrative Officer, DSK-SHIREE, for further inquiries in this regard.

## Abbreviations

CHT: Chittagong Hill Tract
DFID: Department for International Development
DSK: Dushtha Shasthya Kendra
EEP/SHIREE: Economic Empowerment of the Poorest/Stimulating Household Improvements Resulting in Economic Empowerment
ESLEP-CHT: Ensuring Sustainable Livelihood of Extreme Poor of Chittagong Hill Tract
FGD: Focus Group Discussions
GDP: Gross domestic product
GoB: Government of Bangladesh
IDI: In-Depth Interview
KII: Key Informant Interview
NCD: Non-communicable Chronic Diseases
NGO: Non-governmental Organization
UNHCR: United Nations High Commissioner for Refugees

## Footnotes

1 Chittagong Hill Tracts is the only extensively hilly area in south-eastern part of Bangladesh comprises a total area of around 13,295 km^2^ (5,133 sq. mi) which is approximately one-tenth of the total area of Bangladesh. These tracts are encompassed by three hill districts—*Rangamati, Khagrachari* and *Bandarban*. These tracts are the home of over fifteen ethnic groups—approximately 850,000 people.
