# Supplementary material for: Health shocks, care-seeking behaviour and coping strategies of extreme poor households in Bangladesh’s Chittagong Hill tracts
Source: BMC Public Health. 2019 Jul 29;19:1008. doi: 10.1186/s12889-019-7335-7 (PMC6664711; doi:10.1186/s12889-019-7335-7)
Supplement: Supplementary file 1 — of Health shocks, care-seeking behaviour and coping strategies of extreme poor households in Bangladesh’s Chittagong Hill TractsS1_Interview_Guidlines. (DOCX 23 kb) [file 12889_2019_7335_MOESM1_ESM.docx]

Appendix A:

A1: Guideline for In-depth Interview (IDI)

1. Socio-demographic information of the participants (name, age, occupation, education, religion, family structure, income, number and age of household members)
2. Would you please say something about health shock/disease? (What is meant by shock/disease?)
3. What types of health shock/disease have you/your family members experienced/would you please describe your sufferings/pains?
4. Whom did you contact in the process of seeking care? How and who assisted you in the process of care seeking? Who got preference in the care seeking process (Please discuss when, how, why and why not?)
5. What strategies/action did you use to cope up with this shocks/disease? How did you manage these courses of action? (Please discuss elaborately when, how, why and why not?)
6. In your opinion, how and to what extent shock/disease affect household well-being, livelihood support, and/or income generation activities, economic status? (Please discuss when, how, why and why not?)

A2: Guideline for Focused Group Discussion (FGD)

1. Socio-demographic information of the participants (name, age, occupation, education, religion, income, support received, number and age of household members)
2. To what extent the economic condition of you household has changed since the intervention of EEP/Shiree? (How, why, and why not?)
3. Did your household face any health shocks/disease during EEP/Shiree intervention? If yes, what type of health shocks/diseases? What was the initiative? Who was involved in managing these shocks? (Please discuss when, how, why and why not?)
4. In case of health shocks/diseases, whom did you contact in the process of seeking care? How and who assisted you in the process of care seeking? Who got preference in the care seeking process (Please discuss when, how, why and why not?)
5. What strategies/action did you use to cope up with this shocks/disease? How did you manage these courses of action? (Please discuss when, how, why and why not?)
6. In your opinion, how and to what extent have shock/disease affect household well-being, livelihood support, and/or income generation activities, economic status? (Please discuss elaborately when, how, why and why not?)
7. In your opinion, what can be done to improve these conditions? (Why and why not?)

Guideline for Key Informant Interview (KII)

1. In your opinion how is the overall health condition of EEP/Shiree beneficiaries? Why and how did that happen?
2. Please tell us about the EEP/Shiree beneficiaries’ health seeking behavior. Where and why do they prefer to seek care /or why not?
3. Does ethnicity and sparsely dispersed location affect access to and use of health care services (Please check if they mention anything ethnicity, religion etc.)? Why and/or why not?
4. Whether and how does the economic condition of ethnic minority cause failure and/or success in gaining good health (Please check if they mention anything about ethnicity, income, job/occupation, saving, cash, productive assets etc.) Why and/or why not?
5. Others associated factors that cause failure and/or success poor health outcome of ethnic minorities (Please check if they mention anything NGO intervention, policy formulation, program design and implementation, health service governance, etc.) Why and/or why not?
6. In your opinion, what would be the potential measures for improving existing situation and/or household resilience? Why and why not?
